# Non-Communicable Disease Clinical Practice Guidelines in Brazil: A Systematic Assessment of Methodological Quality and Transparency

**DOI:** 10.1371/journal.pone.0166367

**Published:** 2016-11-15

**Authors:** Caroline de Godoi Rezende Costa Molino, Nicolina Silvana Romano-Lieber, Eliane Ribeiro, Daniela Oliveira de Melo

**Affiliations:** 1 Department of Pharmacy, Faculty of Pharmaceutical Sciences, University of São Paulo, São Paulo, Brazil; 2 Department of Public Health Practice, School of Public Health, University of Sao Paulo, São Paulo, Brazil; 3 University of São Paulo Hospital, Department of Pharmacy, Faculty of Pharmaceutical Sciences, University of São Paulo, São Paulo, Brazil; 4 Department of Biological Sciences, Institute of Environmental Sciences, Chemical and Pharmaceutical, Federal University of São Paulo, Diadema, São Paulo, Brazil; Universiti Sains Malaysia, MALAYSIA

## Abstract

**Background:**

Annually, non-communicable diseases (NCDs) kill 38 million people worldwide, with low and middle-income countries accounting for three-quarters of these deaths. High-quality clinical practice guidelines (CPGs) are fundamental to improving NCD management. The present study evaluated the methodological rigor and transparency of Brazilian CPGs that recommend pharmacological treatment for the most prevalent NCDs.

**Methods:**

We conducted a systematic search for CPGs of the following NCDs: asthma, atrial fibrillation, benign prostatic hyperplasia, chronic obstructive pulmonary disease, congestive heart failure, coronary artery disease and/or stable angina, dementia, depression, diabetes, gastroesophageal reflux disease, hypercholesterolemia, hypertension, osteoarthritis, and osteoporosis. CPGs comprising pharmacological treatment recommendations were included. No language or year restrictions were applied. CPGs were excluded if they were merely for local use and referred to NCDs not listed above. CPG quality was independently assessed by two reviewers using the Appraisal of Guidelines Research and Evaluation instrument, version II (AGREE II).

**Main Findings:**

“Scope and purpose” and “clarity and presentation” domains received the highest scores. Sixteen of 26 CPGs were classified as low quality, and none were classified as high overall quality. No CPG was recommended without modification (77% were not recommended at all). After 2009, 2 domain scores (“rigor of development” and “clarity and presentation”) increased (61% and 73%, respectively). However, “rigor of development” was still rated < 30%.

**Conclusion:**

Brazilian healthcare professionals should be concerned with CPG quality for the treatment of selected NCDs. Features that undermined AGREE II scores included the lack of a multidisciplinary team for the development group, no consideration of patients’ preferences, insufficient information regarding literature searches, lack of selection criteria, formulating recommendations, authors’ conflict of interest disclosures, and funding body influence.

## Introduction

Non-communicable diseases (NCDs) are considered major public health problems, strongly affecting the poorest and most vulnerable population groups [1,2]. NCDs kill 38 million people worldwide each year, and 28 million of these deaths occur in low and middle-income countries. Due to NCD healthcare burdens, the World Health Organization (WHO) has emphasized a need for improving NCD treatment [1–3].

High-quality clinical practice guidelines (CPGs) are fundamental for improving healthcare management, as they are special tools that translate scientific research findings, provide explicit recommendations, and support evidence-based decision making [4,5]. CPG implementation is essential for improving NCD control and patient quality of life, as well as optimizing drug utilization and healthcare resources [6–8]. However, several studies have identified CPGs that suffer from low to moderate quality, which call into question the reliability of such measures among healthcare professionals and managers [9–18].

Several tools have been developed worldwide for evaluating CPG quality [19,20]. For instance, the Appraisal of Guidelines Research and Evaluation instrument [21] version II (AGREE II), published in 2009, has been extensively used, validated in several languages, and covers essential information for comprehensive CPG evaluation [19,20]. Several studies worldwide have been conducted for assessing CPG quality using the AGREE II [9–11,16–18,22–25]; however, very little is known regarding CPG quality among low income countries [10]. To date, only one study has evaluated CPG quality in Brazil [18], and none have critically assessed CPGs for NCD treatment quality within a Brazilian sample. Thus, the present study evaluated the methodological rigor and transparency of Brazilian CPGs that recommend pharmacological treatment for the most prevalent NCDs.

## Methods

### Search

The following chronic conditions were selected: asthma, atrial fibrillation, benign prostatic hyperplasia, chronic obstructive pulmonary disease, congestive heart failure, coronary artery disease and/or stable angina, dementia, depression, diabetes, gastroesophageal reflux disease, hypercholesterolemia, hypertension, osteoarthritis, and osteoporosis. These conditions were selected given their high observed prevalence within primary care settings [26–34]. We also included CPGs for rheumatic arthritis and Alzheimer´s disease given their high relevance for elderly adults.

We conducted a comprehensive literature search on October 30, 2015: MEDLINE (by PubMed) [35], LILACS (by Virtual Health Library website) [36], and Cochrane Library [37]. On September 9, 2015, we searched the National Guideline Clearinghouse [38] database. S1 Table contains the full search strategies used for all databases.

We also searched Google for the following (on September 9, 2015): “clinical guidelines” or “therapeutic guidelines” or “clinical protocol” or “clinical practice guidelines” and “Brazil” for each NCD. Finally, we searched for CPGs on the Brazilian Ministry of Health website (on October 31, 2015) available at: http://portalsaude.saude.gov.br/index.php/o-ministerio/principal/leia-mais-o-ministerio/840-sctie-raiz/daf-raiz/cgceaf-raiz/cgceaf/l3-cgceaf/11646-pcdt.

### Guideline selection

A CPG was defined according to the Institute of Medicine: “*clinical practice guidelines are statements that include recommendations intended to optimize patient care that are informed by a systematic review of evidence and an assessment of the benefits and harms of alternative care options*” ([39], p4). Inclusion criteria were CPGs that comprised pharmacological treatment recommendations. No language or year restrictions were applied. Exclusion criteria included CPGs without pharmacological treatment recommendations, were only for local use, and/or referred to chronic conditions not already mentioned.

Two independent reviewers first assessed paper titles and abstracts for potential eligibility. Second, reviewers independently screened each full-text article for inclusion criteria. Finally, we checked medical society and Brazilian Ministry of Health websites for any supplemental material. Discrepancies at any stage were resolved through discussion between the two reviewers. When necessary, a third reviewer was included.

### Data extraction and quality appraisal

One reviewer performed the data extraction. Next, a second reviewer checked the extraction. Included CPGs were assessed according to specific NCDs, publication year, publisher, guideline type (formulated or adapted), guideline references, funding, and quality appraisal.

CPG quality was assigned using the AGREE II instrument [21]. This instrument was chosen because it is a validated tool in Brazil [40] and is widely used [10,20,41]. The AGREE II comprises 23 items, which are grouped into 6 domains, as well as 2 overall assessment items that allow reviewers to rate overall CPG quality and recommend its use [42]. Items are rated on a 7-point scale: 1 (strongly disagree; there is no relevant information or the concept is poorly described) to 7 (strongly agree; quality of information is excellent and all criteria listed in the AGREE II User’s Manual are met) [21]. Reviewers judged the overall CPG quality taking into account all 23 items in accordance with the AGREE II User’s Manual [21].

Two independent reviewers (CGRCM and DOM) assessed CPG quality. The reviewers were trained on the AGREE II instrument as described in S1 Appendix. The reviewers had not previously authored any CPGs. Discrepancies in ratings were verified by calculating Kappa coefficients using VassarStats [43] for each CPG. Kappa coefficients were considered discrepant when ≤0.4, based on Landis and Koch’s criteria [44]. For discrepant CPGs, we identified domains that revealed high discrepancy using a concordance calculator developed by McMaster University [45]. The reviewers then discussed and independently reviewed high discrepant domains. Finally, domain scores were calculated according to the AGREE II User’s Manual.

### Analyses

According to the AGREE II User’s Manual, domain scores must not be calculated as a single quality score. The scores of each domain are determined by the total item score percentage with reference to the range between maximum and minimum scores of that particular domain [42].

Despite having no validated method for an overall classification [42], we decided to adopt a metric for overall CPG quality, as shown in S1 Fig. We prioritized domain 3 for classifying the overall quality since this domain evaluates methodological rigor during CPG development. Other studies have used different classification metrics for determining overall quality [16,46,47]. Then, to differentiate CPGs’ quality regarding other domain scores, we divided it into high, moderate, and low categories with A to C grading. Thus, overall quality was divided into 9 types according to domain 3 and 2 other domains scores (S1 Fig).

Mann-Whitney test was used to test significant difference in domain scores between CPG published before and in/after 2009 (AGREE II publication year). P-values less than 0.05 were considered statistically significant.

## Results

**Fig 1:**
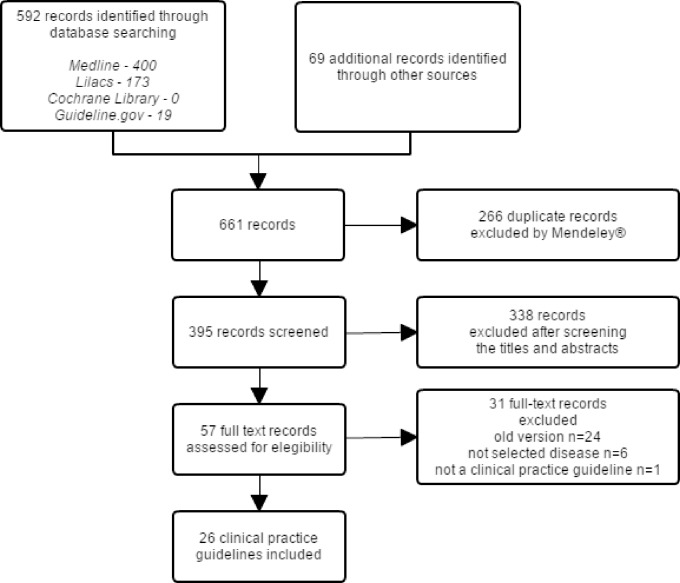
Flowchart of clinical practice guidelines selection.

The search strategy retrieved 661 records, of which 58 were considered for full-text screening; 26 met our eligibility criteria and were assessed using the AGREE II (Fig 1). See S2 Table for excluded CPG details.

Most of the included CPGs were published in/after 2009 (85%). None conducted a systematic review of the literature, explicitly declared funding sources, or claimed to be an adapted CPG. Table 1 shows general characteristics and AGREE II scores of the included CPGs. Few CPGs were published by the Ministry of Health (6; 23%). Regarding the chronic conditions reviewed, only guidelines for dementia treatment were not found. Diabetes mellitus CPGs were the most common. Only two domains (“scope and purpose” and “clarity and presentation”) obtained scores greater than 60%. Most domains scored less than 30%.

**Table 1:** Characteristics of 26 Brazilian CPGs for the treatment of NCDs and AGREE II scores for each CPG. Domain scores >60% are highlighted in bold and CPGs are sorted according to domain 3 score (from highest to lowest in each disease category).

Chronic condition	Publisher	Year of publication	Domain 1	Domain 2	Domain 3	Domain 4	Domain 5	Domain 6	Overall Guideline Assessment	
			Scope and purpose	Stakeholder involvement	Rigor of development	Clarity and presentation	Applicability	Editorial independence	Overall rating	Would reviewers recommend?
Alzheimer´s disease	Ministry of Health[48]	2013	**72%**	42%	42%	**67%**	31%	42%	**67%**	Yes with modification
	Brazilian Medical Association [49]	2011	**61%**	25%	30%	47%	2%	38%	33%	No
Asthma	Ministry of Health [50]	2013	**72%**	36%	41%	**81%**	33%	42%	**67%**	Yes with modification
	Medical Societies [51]	2012	42%	47%	20%	**86%**	42%	21%	33%	No
Atrial fibrillation	Medical Societies [52]	2009	3%	8%	19%	**100%**	25%	29%	17%	No
Benign prostatic hyperplasia	Brazilian Medical Association [53]	2006	36%	22%	18%	53%	10%	42%	25%	No
Chronic obstructive pulmonary disease	Ministry of Health [54]	2013	**72%**	42%	42%	**81%**	33%	42%	58%	Yes with modification
	Brazilian Medical Association [55]	2012	56%	8%	26%	58%	10%	33%	33%	No
	Medical Societies [56]	2004	28%	17%	11%	42%	27%	0%	0%	No
Congestive heart failure	Medical Societies [57]	2012	39%	3%	30%	**67%**	13%	46%	33%	No
Coronary artery disease	Medical Societies [58]	2014	**61%**	33%	24%	**86%**	31%	21%	17%	No
Depression	Brazilian Medical Association [59]	2011	**64%**	22%	24%	47%	2%	38%	25%	No
Diabetes	Medical Societies [60]	2015	33%	3%	34%	**100%**	40%	17%	33%	No
	Medical Societies [61]	2014	58%	8%	23%	**83%**	23%	29%	17%	No
	Brazilian Medical Association [62]	2011	**75%**	39%	22%	**75%**	35%	17%	33%	No
	Brazilian Medical Association [63]	2004	**78%**	14%	21%	39%	2%	17%	25%	No
Gastroesophageal reflux disease	Brazilian Medical Association [64]	2011	33%	17%	21%	44%	0%	38%	25%	No
Hypercholesterolemia	Ministry of Health [65]	2013	**72%**	42%	41%	**75%**	31%	42%	58%	Yes with modification
	Medical Societies [66]	2013	50%	6%	16%	36%	17%	4%	8%	No
Hypertension	Brazilian Medical Association [67]	2004	31%	8%	20%	28%	8%	54%	25%	No
	Medical Societies [68]	2010	11%	11%	18%	**86%**	35%	46%	25%	No
Osteoarthritis	Brazilian Medical Association [69]	2011	39%	22%	27%	**61%**	0%	33%	25%	No
Osteoporosis	Ministry of Health [70]	2014	**72%**	42%	43%	**81%**	40%	42%	**67%**	Yes with modification
	Brazilian Medical Association [71]	2011	**86%**	11%	18%	42%	2%	38%	25%	No
Rheumatoid arthritis	Ministry of Health [72]	2015	**72%**	33%	45%	**78%**	38%	29%	58%	Yes with modification
	Medical Societies [73]	2012	36%	17%	30%	**75%**	42%	21%	25%	No

**Fig 2:**
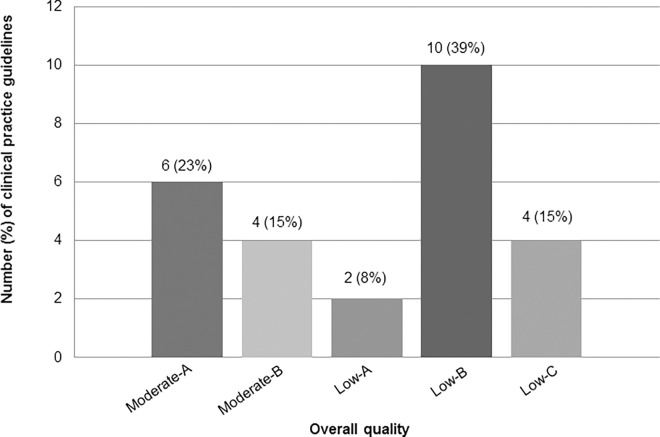
Overall quality classification of Brazilian CPGs (n = 26) for the treatment of the most prevalent NCDs.

Fig 2 shows that most CPGs were classified as low quality (16; 62%). None were classified as high quality.

In terms of whether reviewers would recommend implementing the CPG, we observed that none should be recommended without modifications, and 77% should not be recommended at all. Only CPGs from the Ministry of Health were recommended contingent on modifications.

**Fig 3:**
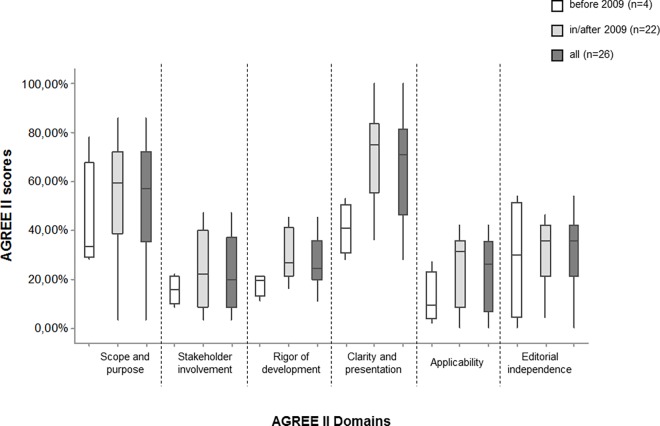
AGREE II scores obtained, per domain, based on publication year (before and in/after 2009, publication of AGREE II) for Brazilian CPGs. (*) Statistical significant was observed for “rigor of development” (p = 0.03) and “clarity and presentation (p = 0.01).”

Improvement in domain scores was observed when comparing CPGs published before and in/after 2009 (Fig 3). However, only 2 domain scores (“rigor of development” and “clarity and presentation”) demonstrated a significant improvement (p < 0.05).

## Discussion

We performed a systematic search of Brazilian CPGs for treating the most prevalent NCDs within primary care settings. Except for dementia, we identified 26 CPGs, with diabetes mellitus as the most common. Most CPGs had been created in the last 5 years; yet, none were of particularly high quality, and most were not recommended by our reviewers. Specifically, 62% of the selected CPGs were deemed “low quality”: low-A (8%), low-B (39%), and low-C (15%). In essence, most of the CPGs scored less than 30% on the “rigor of development” domain. This domain evaluated how evidence for the CPG was assembled and synthesized, how recommendations were formulated, and how the CPG would be updated. Given the deficiencies within this metric, we can conclude poor quality of our selected CPGs. Similar to our findings, Chinese CPGs have been evaluated for poor quality, particularly presenting low scores on the “rigor of development” domain [74–77].

Another important finding was that CPGs published in/after 2009 (AGREE II publication) were rated more positively within 2 domains: “rigor of development” and “clarity and presentation.” One prior study evaluating CPGs published between 1980 and 2007 observed improvement over time in most domains [10]. However, domain scores were still moderate to low [10]. In contrast to these findings, “scope and purpose,” “stakeholder involvement,” and “applicability” domains did not improve post-2009 in the present study. These differences might be explained, in part, by sample size issues, country of origin, and publication year. While we only evaluated 26 Brazilian CPGs, the aforementioned study assessed 42 CPGs across different continents. Additionally, we believe that international CPGs obtain greater scores because the AGREE instrument has been used worldwide since 2003 [20]. The first AGREE version was not validated in Brazil, and AGREE II was not available until 2009. It is interesting to note that only Ministry of Health guidelines were recommended by our reviewers. This result may be explained by the fact that the Ministry of Health guidelines presented better scores in the “rigor of development” domain, with a minimum of 41%. In addition, these guidelines reported AGREE use for developing CPGs, which may be associated with higher quality when compared to others.

Independent from publication year, “scope and purpose” and “clarity and presentation” were more highly rated; however, there is still room for improvement within these domains owing to the presence of scores lower than 30% and between 30 and 60%. As previously reported [10,17], these domains can be ameliorated by describing the target population and health questions, providing clear summaries, and standardizing the CPG presentation form.

“Stakeholder involvement” and “applicability” were the lowest scoring domains, accruing scores < 30%. Other studies [9,10,17,75] have also reported poor quality among these domains. We observed that the “stakeholder involvement” domain did not include consideration of patients’ perspectives, and developer groups were not multidisciplinary. With respect to the “applicability’ domain,” most CPGs did not evaluate resource implications and organizational barriers when applying recommendations, which has also been mentioned previously [9,10,17,75].

Several barriers affect physician adherence to CPG recommendations [78]. CPG developers should explore alternative tools for promoting adherence among healthcare providers. Tools, such as implementation instructions, flowcharts, digital books, and an outline of key recommendations, might be useful for promoting and disseminating CPGs [79]. This should improve “applicability” scores. Additionally, actions toward intensifying healthcare professionals’ participation in CPG development may also improve recommendation adherence [80,81], along with important domain scores. In Brazil, most CPG developers did not create multidisciplinary teams. For instance, specific medical societies only included specialty physicians, while the Brazilian Medical Association included some physicians from different expertise areas. Only the Ministry of Health included a multidisciplinary team and conducted public consultations. Furthermore, insufficient methods for procuring stakeholder views were reported. For instance, only the Ministry of Health’s guidelines conducted public consultations during the final step of guideline development. Few CPGs have conducted a literature search on stakeholder views. More effective CPG creation would likely include patients and patient representatives within the development group [82]. In accordance with our findings, a prior study analyzed 100 endocrine CPGs from North America and demonstrated that only 3 considered patient perspectives and included patients during CPG development [9].

It is interesting to note that all 26 CPGs reviewed presently were undermined by poor rigor of development, even when only analyzing guidelines published in/after 2009. Possible explanations for this result include: 1) no study performed a systematic review or adapted a high-quality CPG; 2) few detailed a specific search strategy and selection criteria; 3) few described the evidence considered and recommendations based on that evidence; and 4) a GRADE system was not used for most of the CPG recommendations. These problems directly affected the “rigor of development” score and overall CPG quality and reliability. Previous reports have noted that most CPGs do not perform a systematic literature search [10,83], as this can be very time-consuming. The AGREE II does not require a systematic review for a full score domain; however, to improve upon specific domain scores (namely “rigor of development,”), CPGs must explicitly describe their selection criteria and the type of evidence considered [42]. Low “rigor of development” scores might also be related to the various CPG versions that have been published (digital versions as eBooks, full vs. summary versions, etc.) [18]. For instance, the Ministry of Health provides partial methods sections and mentions specific CPG books. Medical societies often publish CPGs in scientific journals and digital books comprising flowcharts and recommendation summaries; however, most do not present a methods section. The Brazilian Medical Association provides digital books, including a general methods section for all CPGs published. Yet, no information regarding criteria selection and the formulation of recommendations is provided.

The “editorial independence” median score was 35.5% (interquartile range, 21–42%). Although most CPGs disclose authors’ competing interests, they did not address how identified conflicts would be managed. Moreover, none clearly disclosed funding sources. Thus, these results need to be interpreted with caution. AGREE II scores rely on CPG text; therefore, we believe that competing interests were properly assessed. A more careful description was lacking. Furthermore, low scores within this domain have been reported elsewhere [84]. Scores range from 16% among Chinese CPGs for hypertension to 30% for a separate analysis of 626 CPGs, and 41% for European CPGs specific to chronic diseases [10,17,75]. Moreover, a review of 250 CPGs observed the absence of author disclosures (40%), unavailable author disclosures within the public domain (42%), and at least 60% provided at least one author with a conflict [84].

In order to improve CPG quality, we suggest focusing on the following: 1) assembling a multidisciplinary development group; 2) take into account patients’ preferences; 3) describe literature search details, selection criteria, and the formulation of recommendations; and 4) explicitly declare any competing or financial interests among the authors. Finally, we believe high-quality CPGs will likely require a partnership between health institutions and universities with CPG development expertise. Previous work in Saudi Arabia has suggested that collaborative work between the Ministry of Health and McMaster University enabled the production of 10 CPGs, with 80 recommendations, within 4 months [85]. Thus, knowing that the Brazilian health system is a reference in Latin America, the Caribbean, and Portuguese-speaking African countries, the study of Brazilian CPGs is essential for healthcare professionals in many other countries. Health institutions should identify universities with CPG expertise and work collaboratively toward promoting high-quality CPG development and adaptation.

### Limitations and strengths

To date, this is the first study to assess Brazilian CPGs’ quality for the most prevalent NCDs, as well as conduct a comprehensive search for identifying Brazilian guidelines. Prior Brazilian research evaluated only 8 Ministry of Health guidelines, and most were for managing rare diseases [18]. Nevertheless, the present study is limited by a subjective analysis of the AGREE II instrument. However, raters received exhaustive training on the instrument, which should ameliorate assessment concerns [21].

## Conclusions

The present study evaluated Brazilian CPGs for treating the most prevalent NCDs. Our results revealed an urgent need to improve CPG development in Brazil. Healthcare professionals should be concerned with current CPG quality given that most were classified as low-B, suggesting poor “rigor of development” and less than stellar scores across additional domains. Overall, it is recommended that health institutions work in partnership with universities and adopt the AGREE II for improving CPGs’ quality.

## Supporting Information

S1 Appendix Reviewers training in AGREE II instrument. (DOCX)

S1 Fig Overall quality classification for each CPG. (TIF)

S1 Table Systematic search strategies. (DOCX)

S2 Table Excluded CPG information. (DOCX)

S3 Table PRISMA Checklist. (DOC)
