# Supplementary material for: Non-Communicable Disease Clinical Practice Guidelines in Brazil: A Systematic Assessment of Methodological Quality and Transparency
Source: PLoS One. 2016 Nov 15;11(11):e0166367. doi: 10.1371/journal.pone.0166367 (PMC5112889; doi:10.1371/journal.pone.0166367)
Supplement: S1 Appendix — (DOCX) [file pone.0166367.s001.docx]

**S1 Appendix. Reviewers training in AGREE II instrument.**

Reviewers, both clinical pharmacists, were trained in AGREE II instrument using the online tutorial available at <http://www.agreetrust.org/>. In addition, both reviewers had completed the CPG course provided by the Pan American Health Organization/ WHO and the Brazilian Ministry of Health (<http://www.aulas.cvspbrasil.fiocruz.br/>).

Prior to evaluating selected CPGs, reviewers independently evaluated 3 other CPGs: Gaucher disease, primary dysmenorrhea, and chronic pain. All discrepancies were discussed between the reviewers and a specialist in AGREE instrument.
