# Supplementary figures and images for: Non-Communicable Disease Clinical Practice Guidelines in Brazil: A Systematic Assessment of Methodological Quality and Transparency
Source: PLoS One. 2016 Nov 15;11(11):e0166367. doi: 10.1371/journal.pone.0166367 (PMC5112889; doi:10.1371/journal.pone.0166367)

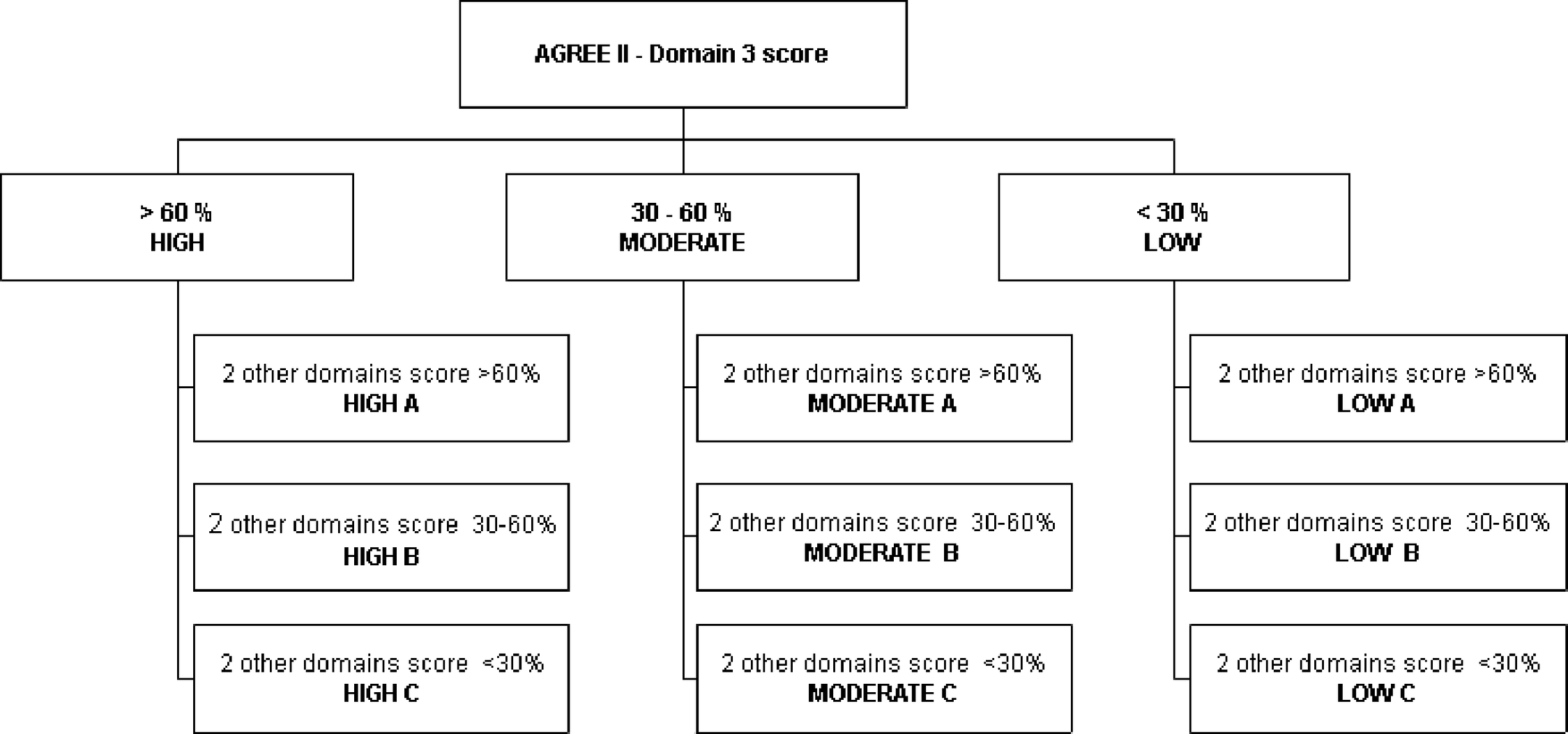

Supplement: S1 Fig — (TIF) [file pone.0166367.s002.tif]
