# Supplementary material for: Non-Communicable Disease Clinical Practice Guidelines in Brazil: A Systematic Assessment of Methodological Quality and Transparency
Source: PLoS One. 2016 Nov 15;11(11):e0166367. doi: 10.1371/journal.pone.0166367 (PMC5112889; doi:10.1371/journal.pone.0166367)
Supplement: S1 Table — (DOCX) [file pone.0166367.s003.docx]

**S1 Table. Systematic search strategies.**

**Medline (by Pubmed website) at 10/30/2015**

| ((("Guideline" [Publication Type] OR "Guidelines as Topic"[Mesh] OR "Practice Guideline" [Publication Type]) OR "Clinical Protocols"[Mesh]) OR "Consensus"[Mesh]) OR "Standard of Care"[Mesh] OR Protocol, Clinical OR Clinical Protocol OR Protocols, Clinical OR Treatment Protocols OR Treatment Protocol OR Protocols, Treatment OR Clinical Research Protocol OR Research Protocols, Clinical OR Protocols, Clinical Research OR Research Protocol, Clinical OR Clinical Research Protocols OR Protocol, Clinical Research OR Consensus Development OR Development, Consensus OR Care Standard OR Care Standards OR Standards of Care AND "Brazil"[Mesh] AND "Hypertension"[Mesh] OR Blood Pressure, High OR Blood Pressures, High OR High Blood Pressure OR High Blood Pressures) |
| --- |
| ((("Guideline" [Publication Type] OR "Guidelines as Topic"[Mesh] OR "Practice Guideline" [Publication Type]) OR "Clinical Protocols"[Mesh]) OR "Consensus"[Mesh]) OR "Standard of Care"[Mesh] OR Protocol, Clinical OR Clinical Protocol OR Protocols, Clinical OR Treatment Protocols OR Treatment Protocol OR Protocols, Treatment OR Clinical Research Protocol OR Research Protocols, Clinical OR Protocols, Clinical Research OR Research Protocol, Clinical OR Clinical Research Protocols OR Protocol, Clinical Research OR Consensus Development OR Development, Consensus OR Care Standard OR Care Standards OR Standards of Care AND "Brazil"[Mesh] AND "Heart Failure"[Mesh] OR Cardiac Failure OR Heart Decompensation OR Decompensation, Heart OR Heart Failure, Right-Sided OR Heart Failure, Right Sided OR Right-Sided Heart Failure OR Right Sided Heart Failure OR Myocardial Failure OR Congestive Heart Failure OR Heart Failure, Congestive OR Heart Failure, Left-Sided OR Heart Failure, Left Sided OR Left-Sided Heart Failure OR Left Sided Heart Failure) |
| ((("Guideline" [Publication Type] OR "Guidelines as Topic"[Mesh] OR "Practice Guideline" [Publication Type]) OR "Clinical Protocols"[Mesh]) OR "Consensus"[Mesh]) OR "Standard of Care"[Mesh] OR Protocol, Clinical OR Clinical Protocol OR Protocols, Clinical OR Treatment Protocols OR Treatment Protocol OR Protocols, Treatment OR Clinical Research Protocol OR Research Protocols, Clinical OR Protocols, Clinical Research OR Research Protocol, Clinical OR Clinical Research Protocols OR Protocol, Clinical Research OR Consensus Development OR Development, Consensus OR Care Standard OR Care Standards OR Standards of Care AND "Brazil"[Mesh] AND "Angina, Stable"[Mesh] OR Anginas, Stable OR Stable Angina OR Stable Anginas OR Chronic Stable Angina OR Angina, Chronic Stable OR Anginas, Chronic Stable OR Chronic Stable Anginas OR Stable Angina, Chronic OR Stable Anginas, Chronic OR Angina Pectoris, Stable OR Angina Pectori, Stable OR Pectori, Stable Angina OR Pectoris, Stable Angina OR Stable Angina Pectori OR Stable Angina Pectoris) |
| ((("Guideline" [Publication Type] OR "Guidelines as Topic"[Mesh] OR "Practice Guideline" [Publication Type]) OR "Clinical Protocols"[Mesh]) OR "Consensus"[Mesh]) OR "Standard of Care"[Mesh] OR Protocol, Clinical OR Clinical Protocol OR Protocols, Clinical OR Treatment Protocols OR Treatment Protocol OR Protocols, Treatment OR Clinical Research Protocol OR Research Protocols, Clinical OR Protocols, Clinical Research OR Research Protocol, Clinical OR Clinical Research Protocols OR Protocol, Clinical Research OR Consensus Development OR Development, Consensus OR Care Standard OR Care Standards OR Standards of Care AND "Brazil"[Mesh] AND "Atrial Fibrillation"[Mesh]OR Atrial Fibrillations OR Fibrillation, Atrial OR Fibrillations, Atrial OR Familial Atrial Fibrillation OR Auricular Fibrillation OR Auricular Fibrillations OR Fibrillation, Auricular OR Fibrillations, Auricular |
| ((("Guideline" [Publication Type] OR "Guidelines as Topic"[Mesh] OR "Practice Guideline" [Publication Type]) OR "Clinical Protocols"[Mesh]) OR "Consensus"[Mesh]) OR "Standard of Care"[Mesh] OR Protocol, Clinical OR Clinical Protocol OR Protocols, Clinical OR Treatment Protocols OR Treatment Protocol OR Protocols, Treatment OR Clinical Research Protocol OR Research Protocols, Clinical OR Protocols, Clinical Research OR Research Protocol, Clinical OR Clinical Research Protocols OR Protocol, Clinical Research OR Consensus Development OR Development, Consensus OR Care Standard OR Care Standards OR Standards of Care AND "Brazil"[Mesh] AND "Coronary Artery Disease"[Mesh] OR Artery Disease, Coronary OR Artery Diseases, Coronary OR Coronary Artery Diseases OR Disease, Coronary Artery OR Diseases, Coronary Artery OR Coronary Arteriosclerosis OR Arterioscleroses, Coronary OR Coronary Arterioscleroses OR Atherosclerosis, Coronary OR Atheroscleroses, Coronary OR Coronary Atheroscleroses OR Coronary Atherosclerosis OR Arteriosclerosis, Coronary) |
| ((("Guideline" [Publication Type] OR "Guidelines as Topic"[Mesh] OR "Practice Guideline" [Publication Type]) OR "Clinical Protocols"[Mesh]) OR "Consensus"[Mesh]) OR "Standard of Care"[Mesh] OR Protocol, Clinical OR Clinical Protocol OR Protocols, Clinical OR Treatment Protocols OR Treatment Protocol OR Protocols, Treatment OR Clinical Research Protocol OR Research Protocols, Clinical OR Protocols, Clinical Research OR Research Protocol, Clinical OR Clinical Research Protocols OR Protocol, Clinical Research OR Consensus Development OR Development, Consensus OR Care Standard OR Care Standards OR Standards of Care AND "Brazil"[Mesh] AND "Hypercholesterolemia"[Mesh] OR Hypercholesterolemias OR Elevated Cholesterol OR Hypercholesteremia OR Hypercholesteremias) |
| ((("Guideline" [Publication Type] OR "Guidelines as Topic"[Mesh] OR "Practice Guideline" [Publication Type]) OR "Clinical Protocols"[Mesh]) OR "Consensus"[Mesh]) OR "Standard of Care"[Mesh] OR Protocol, Clinical OR Clinical Protocol OR Protocols, Clinical OR Treatment Protocols OR Treatment Protocol OR Protocols, Treatment OR Clinical Research Protocol OR Research Protocols, Clinical OR Protocols, Clinical Research OR Research Protocol, Clinical OR Clinical Research Protocols OR Protocol, Clinical Research OR Consensus Development OR Development, Consensus OR Care Standard OR Care Standards OR Standards of Care AND "Brazil"[Mesh] AND "Diabetes Mellitus"[Mesh] OR "Diabetes Mellitus, Type 2"[Mesh]OR Diabetes Insipidus OR Diabetic Diet OR Prediabetic State OR Scleredema Adultorum OR Glycosylation End Products, Advanced OR Glucose Intolerance OR Gastroparesis OR NIDDM OR Maturity-Onset Diabetes OR Diabetes Mellitus, Noninsulin-Dependent OR Diabetes Mellitus, Adult-Onset OR Adult-Onset Diabetes Mellitus OR Diabetes Mellitus, Adult Onset OR Diabetes Mellitus, Ketosis-Resistant OR Diabetes Mellitus, Ketosis Resistant OR Ketosis-Resistant Diabetes Mellitus OR Diabetes Mellitus, Maturity-Onset OR Diabetes Mellitus, Maturity Onset OR Diabetes Mellitus, Non Insulin Dependent OR Diabetes Mellitus, Non-Insulin-Dependent OR Non-Insulin-Dependent Diabetes Mellitus OR Diabetes Mellitus, Noninsulin Dependent OR Diabetes Mellitus, Slow-Onset OR Diabetes Mellitus, Slow Onset OR Slow-Onset Diabetes Mellitus OR Diabetes Mellitus, Stable OR Stable Diabetes Mellitus OR Diabetes Mellitus, Type II OR Maturity-Onset Diabetes Mellitus OR Maturity Onset Diabetes Mellitus OR MODY OR Type 2 Diabetes Mellitus OR Noninsulin-Dependent Diabetes Mellitus) |
| ((("Guideline" [Publication Type] OR "Guidelines as Topic"[Mesh] OR "Practice Guideline" [Publication Type]) OR "Clinical Protocols"[Mesh]) OR "Consensus"[Mesh]) OR "Standard of Care"[Mesh] OR Protocol, Clinical OR Clinical Protocol OR Protocols, Clinical OR Treatment Protocols OR Treatment Protocol OR Protocols, Treatment OR Clinical Research Protocol OR Research Protocols, Clinical OR Protocols, Clinical Research OR Research Protocol, Clinical OR Clinical Research Protocols OR Protocol, Clinical Research OR Consensus Development OR Development, Consensus OR Care Standard OR Care Standards OR Standards of Care AND "Brazil"[Mesh] AND "Osteoarthritis"[Mesh] OR Osteoarthritides OR Osteoarthrosis OR Osteoarthroses OR Arthritis, Degenerative OR Arthritides, Degenerative OR Degenerative Arthritides OR Degenerative Arthritis OR Osteoarthrosis Deformans) |
| ((("Guideline" [Publication Type] OR "Guidelines as Topic"[Mesh] OR "Practice Guideline" [Publication Type]) OR "Clinical Protocols"[Mesh]) OR "Consensus"[Mesh]) OR "Standard of Care"[Mesh] OR Protocol, Clinical OR Clinical Protocol OR Protocols, Clinical OR Treatment Protocols OR Treatment Protocol OR Protocols, Treatment OR Clinical Research Protocol OR Research Protocols, Clinical OR Protocols, Clinical Research OR Research Protocol, Clinical OR Clinical Research Protocols OR Protocol, Clinical Research OR Consensus Development OR Development, Consensus OR Care Standard OR Care Standards OR Standards of Care AND "Brazil"[Mesh] AND "Osteoporosis"[Mesh] OR Osteoporoses OR Osteoporosis, Post-Traumatic OR Osteoporosis, Post Traumatic OR Post-Traumatic Osteoporoses OR Post-Traumatic Osteoporosis OR Osteoporosis, Senile OR Osteoporoses, Senile OR Senile Osteoporoses OR Senile Osteoporosis OR Osteoporosis, Involutional OR Osteoporosis, Age-Related OR Osteoporosis, Age Related OR Bone Loss, Age-Related OR Age-Related Bone Loss OR Age-Related Bone Losses OR Bone Loss, Age Related OR Bone Losses, Age-Related OR Age-Related Osteoporosis OR Age Related Osteoporosis OR Age-Related Osteoporoses OR Osteoporoses, Age-Related) |
| ((("Guideline" [Publication Type] OR "Guidelines as Topic"[Mesh] OR "Practice Guideline" [Publication Type]) OR "Clinical Protocols"[Mesh]) OR "Consensus"[Mesh]) OR "Standard of Care"[Mesh] OR Protocol, Clinical OR Clinical Protocol OR Protocols, Clinical OR Treatment Protocols OR Treatment Protocol OR Protocols, Treatment OR Clinical Research Protocol OR Research Protocols, Clinical OR Protocols, Clinical Research OR Research Protocol, Clinical OR Clinical Research Protocols OR Protocol, Clinical Research OR Consensus Development OR Development, Consensus OR Care Standard OR Care Standards OR Standards of Care AND "Brazil"[Mesh] AND "Pulmonary Disease, Chronic Obstructive"[Mesh] OR COPD OR Chronic Obstructive Pulmonary Disease OR COAD OR Chronic Obstructive Airway Disease OR Chronic Obstructive Lung Disease OR Airflow Obstruction, Chronic OR Airflow Obstructions, Chronic OR Chronic Airflow Obstructions OR Chronic Airflow Obstruction) |
| ((("Guideline" [Publication Type] OR "Guidelines as Topic"[Mesh] OR "Practice Guideline" [Publication Type]) OR "Clinical Protocols"[Mesh]) OR "Consensus"[Mesh]) OR "Standard of Care"[Mesh] OR Protocol, Clinical OR Clinical Protocol OR Protocols, Clinical OR Treatment Protocols OR Treatment Protocol OR Protocols, Treatment OR Clinical Research Protocol OR Research Protocols, Clinical OR Protocols, Clinical Research OR Research Protocol, Clinical OR Clinical Research Protocols OR Protocol, Clinical Research OR Consensus Development OR Development, Consensus OR Care Standard OR Care Standards OR Standards of Care AND "Brazil"[Mesh] AND "Asthma"[Mesh] OR Asthmas OR Bronchial Asthma OR Asthma, Bronchial) |
| ((("Guideline" [Publication Type] OR "Guidelines as Topic"[Mesh] OR "Practice Guideline" [Publication Type]) OR "Clinical Protocols"[Mesh]) OR "Consensus"[Mesh]) OR "Standard of Care"[Mesh] OR Protocol, Clinical OR Clinical Protocol OR Protocols, Clinical OR Treatment Protocols OR Treatment Protocol OR Protocols, Treatment OR Clinical Research Protocol OR Research Protocols, Clinical OR Protocols, Clinical Research OR Research Protocol, Clinical OR Clinical Research Protocols OR Protocol, Clinical Research OR Consensus Development OR Development, Consensus OR Care Standard OR Care Standards OR Standards of Care AND "Brazil"[Mesh] AND "Gastroesophageal Reflux"[Mesh] OR Gastric Acid Reflux OR Acid Reflux, Gastric OR Reflux, Gastric Acid OR Gastric Acid Reflux Disease OR Gastro-Esophageal Reflux OR Gastro Esophageal Reflux OR Reflux, Gastro-Esophageal OR Gastroesophageal Reflux Disease OR GERD OR Reflux, Gastroesophageal OR Esophageal Reflux OR Gastro-oesophageal Reflux OR Gastro oesophageal Reflux OR Reflux, Gastro-oesophageal) |
| ((("Guideline" [Publication Type] OR "Guidelines as Topic"[Mesh] OR "Practice Guideline" [Publication Type]) OR "Clinical Protocols"[Mesh]) OR "Consensus"[Mesh]) OR "Standard of Care"[Mesh] OR Protocol, Clinical OR Clinical Protocol OR Protocols, Clinical OR Treatment Protocols OR Treatment Protocol OR Protocols, Treatment OR Clinical Research Protocol OR Research Protocols, Clinical OR Protocols, Clinical Research OR Research Protocol, Clinical OR Clinical Research Protocols OR Protocol, Clinical Research OR Consensus Development OR Development, Consensus OR Care Standard OR Care Standards OR Standards of Care AND "Brazil"[Mesh] AND "Dementia"[Mesh] OR Dementias OR Amentia OR Amentias OR Senile Paranoid Dementia OR Dementias, Senile Paranoid OR Paranoid Dementia, Senile OR Paranoid Dementias, Senile OR Senile Paranoid Dementias OR Familial Dementia OR Dementia, Familial OR Dementias, Familial OR Familial Dementias) |
| ((("Guideline" [Publication Type] OR "Guidelines as Topic"[Mesh] OR "Practice Guideline" [Publication Type]) OR "Clinical Protocols"[Mesh]) OR "Consensus"[Mesh]) OR "Standard of Care"[Mesh] OR Protocol, Clinical OR Clinical Protocol OR Protocols, Clinical OR Treatment Protocols OR Treatment Protocol OR Protocols, Treatment OR Clinical Research Protocol OR Research Protocols, Clinical OR Protocols, Clinical Research OR Research Protocol, Clinical OR Clinical Research Protocols OR Protocol, Clinical Research OR Consensus Development OR Development, Consensus OR Care Standard OR Care Standards OR Standards of Care AND "Brazil"[Mesh] AND "Depression"[Mesh] OR "Depressive Disorder"[Mesh] OR Depressions OR Depressive Symptoms OR Depressive Symptom OR Symptom, Depressive OR Symptoms, Depressive OR Emotional Depression OR Depression, Emotional OR Depressions, Emotional OR Emotional Depressions OR Depressive Disorders OR Disorder, Depressive OR Disorders, Depressive OR Neurosis, Depressive OR Depressive Neuroses OR Depressive Neurosis OR Neuroses, Depressive OR Depression, Endogenous OR Depressions, Endogenous OR Endogenous Depression OR Endogenous Depressions OR Depressive Syndrome OR Depressive Syndromes OR Syndrome, Depressive OR Syndromes, Depressive OR Depression, Neurotic OR Depressions, Neurotic OR Neurotic Depression OR Neurotic Depressions OR Melancholia OR Melancholias OR Unipolar Depression OR Depression, Unipolar OR Depressions, Unipolar OR Unipolar Depressions) |
| ((("Guideline" [Publication Type] OR "Guidelines as Topic"[Mesh] OR "Practice Guideline" [Publication Type]) OR "Clinical Protocols"[Mesh]) OR "Consensus"[Mesh]) OR "Standard of Care"[Mesh] OR Protocol, Clinical OR Clinical Protocol OR Protocols, Clinical OR Treatment Protocols OR Treatment Protocol OR Protocols, Treatment OR Clinical Research Protocol OR Research Protocols, Clinical OR Protocols, Clinical Research OR Research Protocol, Clinical OR Clinical Research Protocols OR Protocol, Clinical Research OR Consensus Development OR Development, Consensus OR Care Standard OR Care Standards OR Standards of Care AND "Brazil"[Mesh] AND "Prostatic Hyperplasia"[Mesh] OR Hyperplasia, Prostatic OR Prostatic Hypertrophy OR Adenoma, Prostatic OR Adenomas, Prostatic OR Prostatic Adenomas OR Prostatic Adenoma OR Benign Prostatic Hyperplasia OR Prostatic Hyperplasia, Benign OR Prostatic Hypertrophy, Benign OR Benign Prostatic Hypertrophy OR Hypertrophy, Benign Prostatic) |

**LILACS at 10/30/2015**

| MH:"Guia de Prática Clínica" OR MH:"Guía de Práctica Clínica" OR MH:"Practice Guideline" OR MH:V02.515.500$ OR (diretriz terapeutica) OR (therapeutic guideline) OR (directrices terapéuticas) OR (protocolo clinico) OR (clinical protocol) AND MH:Brasil OR MH:Brazil OR MH:Z01.107.757.176$ AND MH:Hipertensão OR MH:Hipertensión OR MH:Hypertension OR MH:C14.907.489$ OR (hipertensão) OR (hipertensão arterial) OR (hipertensão essencial) OR (arterial hypertension) OR (essential hypertension) OR (arterial hipertensión) OR (essential hipertensión) |
| --- |
| MH:"Guia de Prática Clínica" OR MH:"Guía de Práctica Clínica" OR MH:"Practice Guideline" OR MH:V02.515.500$ OR (diretriz terapeutica) OR (therapeutic guideline) OR (directrices terapéuticas) OR (protocolo clinico) OR (clinical protocol) AND MH:Brasil OR MH:Brazil OR MH:Z01.107.757.176$ AND MH:"Insuficiência Cardíaca" OR MH:"Heart Failure" OR MH:C14.280.434$ OR (insuficiencia cardiaca cronica) OR (insuficiencia cardiaca) OR (heart failure) OR (chronic heart failure) OR (falencia cardiaca) OR (insuficiencia cardiaca congestiva) |
| MH:"Guia de Prática Clínica" OR MH:"Guía de Práctica Clínica" OR MH:"Practice Guideline" OR MH:V02.515.500$ OR (diretriz terapeutica) OR (therapeutic guideline) OR (directrices terapéuticas) OR (protocolo clinico) OR (clinical protocol) AND MH:Brasil OR MH:Brazil OR MH:Z01.107.757.176$ AND MH:"Angina Pectoris" OR MH:"Angina de Pecho" OR MH:C14.280.647.187$ OR MH:C14.907.585.187$ OR MH:C23.888.646.215.500$ OR MH:"Angina Estável" OR MH:"Angina Estable" OR MH:"Angina, Stable" OR MH:C14.280.647.187.362$ OR MH:C14.907.585.187.362$ OR MH:C23.888.646.215.500.575$ OR (angina estavel) OR (stable angina) OR (angina estable) |
| MH:"Guia de Prática Clínica" OR MH:"Guía de Práctica Clínica" OR MH:"Practice Guideline" OR MH:V02.515.500$ OR (diretriz terapeutica) OR (therapeutic guideline) OR (directrices terapéuticas) OR (protocolo clinico) OR (clinical protocol) AND MH:Brasil OR MH:Brazil OR MH:Z01.107.757.176$ AND MH:"Fibrilação Atrial" OR MH:"Fibrilación Atrial" OR MH:"Atrial Fibrillation" OR MH:C14.280.067.198$ OR MH:C23.550.073.198$ OR (fibrilação atrial) OR (Atrial Fibrillation) OR (Fibrilación Atrial) |
| MH:"Guia de Prática Clínica" OR MH:"Guía de Práctica Clínica" OR MH:"Practice Guideline" OR MH:V02.515.500$ OR (diretriz terapeutica) OR (therapeutic guideline) OR (directrices terapéuticas) OR (protocolo clinico) OR (clinical protocol) AND MH:Brasil OR MH:Brazil OR MH:Z01.107.757.176$ AND MH:"Doença das Coronárias" OR MH:"Enfermedad Coronaria" OR MH:"Coronary Disease" OR MH:C14.280.647.250$ OR MH:C14.907.585.250$ OR MH:"Doença da Artéria Coronariana" OR MH:"Enfermedad de la Arteria Coronaria" OR MH:"Coronary Artery Disease" OR MH: C14.280.647.250.260$ OR MH:C14.907.137.126.339$ OR MH:C14.907.585.250.260$ OR MH:"Doenças das Artérias Carótidas" OR MH:"Enfermedades de las Arterias Carótidas" OR MH:"Carotid Artery Diseases" OR MH:C10.228.140.300.200$ OR MH:C14.907.253.123$ OR MH:Aterosclerose OR MH:Aterosclerosis OR MH:Atherosclerosis OR MH:C14.907.137.126.307$ |
| MH:"Guia de Prática Clínica" OR MH:"Guía de Práctica Clínica" OR MH:"Practice Guideline" OR MH:V02.515.500$ OR (diretriz terapeutica) OR (therapeutic guideline) OR (directrices terapéuticas) OR (protocolo clinico) OR (clinical protocol) AND MH:Brasil OR MH:Brazil OR MH:Z01.107.757.176$ AND MH: Hipercolesterolemia OR MH:Hypercholesterolemia OR MH:C18.452.584.500.500.396$ OR MH:Dislipidemias OR MH:Dyslipidemias OR MH:C18.452.584.500$ |
| MH:"Guia de Prática Clínica" OR MH:"Guía de Práctica Clínica" OR MH:"Practice Guideline" OR MH:V02.515.500$ OR (diretriz terapeutica) OR (therapeutic guideline) OR (directrices terapéuticas) OR (protocolo clinico) OR (clinical protocol) AND MH:Brasil OR MH:Brazil OR MH:Z01.107.757.176$ AND MH:"Diabetes Mellitus" OR MH:C18.452.394.750$ OR MH:C19.246$ OR MH:"Diabetes Mellitus Tipo 2" OR MH:"Diabetes Mellitus, Type 2" OR MH:C18.452.394.750.149$ OR MH:C19.246.300$ |
| MH:"Guia de Prática Clínica" OR MH:"Guía de Práctica Clínica" OR MH:"Practice Guideline" OR MH:V02.515.500$ OR (diretriz terapeutica) OR (therapeutic guideline) OR (directrices terapéuticas) OR (protocolo clinico) OR (clinical protocol) AND MH:Brasil OR MH:Brazil OR MH:Z01.107.757.176$ AND MH:Osteoartrite OR MH:Osteoartritis OR MH:Osteoarthritis OR MH:C05.550.114.606$ OR MH:C05.799.613$ OR (ARTROSE) OR (OSTEOARTROSE) |
| MH:"Guia de Prática Clínica" OR MH:"Guía de Práctica Clínica" OR MH:"Practice Guideline" OR MH:V02.515.500$ OR (diretriz terapeutica) OR (therapeutic guideline) OR (directrices terapéuticas) OR (protocolo clinico) OR (clinical protocol) AND MH:Brasil OR MH:Brazil OR MH:Z01.107.757.176$ AND MH:Osteoporose OR MH:Osteoporosis OR MH:C05.116.198.579$ |
| MH:"Guia de Prática Clínica" OR MH:"Guía de Práctica Clínica" OR MH:"Practice Guideline" OR MH:V02.515.500$ OR (diretriz terapeutica) OR (therapeutic guideline) OR (directrices terapéuticas) OR (protocolo clinico) OR (clinical protocol) AND MH:Brasil OR MH:Brazil OR MH:Z01.107.757.176$ AND MH:"Doença Pulmonar Obstrutiva Crônica" OR MH:"Enfermedad Pulmonar Obstructiva Crónica" OR MH:"Pulmonary Disease, Chronic Obstructive" OR MH:C08.381.495.389$ |
| MH:"Guia de Prática Clínica" OR MH:"Guía de Práctica Clínica" OR MH:"Practice Guideline" OR MH:V02.515.500$ OR (diretriz terapeutica) OR (therapeutic guideline) OR (directrices terapéuticas) OR (protocolo clinico) OR (clinical protocol) AND MH:Brasil OR MH:Brazil OR MH:Z01.107.757.176$ AND MH:Asma OR MH:Asthma OR MH:C08.127.108$ OR MH:C08.381.495.108$ OR MH:C08.674.095$ OR MH:C20.543.480.680.095$ OR MH:SP4.001.012.143.134$ OR MH:SP4.046.452.698.904.175$ |
| MH:"Guia de Prática Clínica" OR MH:"Guía de Práctica Clínica" OR MH:"Practice Guideline" OR MH:V02.515.500$ OR (diretriz terapeutica) OR (therapeutic guideline) OR (directrices terapéuticas) OR (protocolo clinico) OR (clinical protocol) AND MH:Brasil OR MH:Brazil OR MH:Z01.107.757.176$ AND MH:"Refluxo Duodenogástrico" OR MH:"Reflujo Duodenogástrico" OR MH:"Duodenogastric Reflux" OR MH:C06.405.469.275.700$ OR MH:C06.405.748.240$ OR MH:"Esofagite Péptica" OR MH:"Esofagitis Péptica" OR MH:"Esophagitis, Peptic" OR MH:C06.405.117.620.420$ OR MH:C06.405.205.663.420$ OR MH:C06.405.608.348$ OR MH:"Refluxo Gastroesofágico" OR MH:"Reflujo Gastroesofágico" OR MH:"Gastroesophageal Reflux" OR MH:C06.405.117.119.500.484$ |
| MH:"Guia de Prática Clínica" OR MH:"Guía de Práctica Clínica" OR MH:"Practice Guideline" OR MH:V02.515.500$ OR (diretriz terapeutica) OR (therapeutic guideline) OR (directrices terapéuticas) OR (protocolo clinico) OR (clinical protocol) AND MH:Brasil OR MH:Brazil OR MH:Z01.107.757.176$ AND MH:Demência OR MH:Demencia OR MH:Dementia OR MH:C10.228.140.380$ OR MH:F03.087.400$ |
| MH:"Guia de Prática Clínica" OR MH:"Guía de Práctica Clínica" OR MH:"Practice Guideline" OR MH:V02.515.500$ OR (diretriz terapeutica) OR (therapeutic guideline) OR (directrices terapéuticas) OR (protocolo clinico) OR (clinical protocol) AND MH:Brasil OR MH:Brazil OR MH:Z01.107.757.176$ AND MH:Depressão OR MH:Depresión OR MH:Depression OR MH:F01.145.126.350$ |
| MH:"Guia de Prática Clínica" OR MH:"Guía de Práctica Clínica" OR MH:"Practice Guideline" OR MH:V02.515.500$ OR (diretriz terapeutica) OR (therapeutic guideline) OR (directrices terapéuticas) OR (protocolo clinico) OR (clinical protocol) AND MH:Brasil OR MH:Brazil OR MH:Z01.107.757.176$ AND MH:"Hiperplasia Prostática" OR MH:"Prostatic Hyperplasia" OR MH:C12.294.565.500$ |

**Cochrane Library T 10/30/2015**

| **ID** | **Search** |
| --- | --- |
| #1 | MeSH descriptor: [Guideline] explode all trees |
| #2 | MeSH descriptor: [Consensus] explode all trees |
| #3 | MeSH descriptor: [Practice Guideline] explode all trees |
| #4 | #1 or #2 or #3 |
| #5 | MeSH descriptor: [Brazil] explode all trees |
| #6 | #4 and #5 |

**National Guideline Clearinghouse (guidelines.gov) at 11/09/2015**

[Advanced Search](http://www.guideline.gov/search/advanced-search.aspx) >

Keyword: Brazil
Guideline Category: Treatment

**Google website at 11/09/2015**

Terms: clinical guideline or therapeutic guidelines or clinical protocol or clinical practice guideline and Brazil and each of 15 NCDs listed
