# Supplementary material for: Non-Communicable Disease Clinical Practice Guidelines in Brazil: A Systematic Assessment of Methodological Quality and Transparency
Source: PLoS One. 2016 Nov 15;11(11):e0166367. doi: 10.1371/journal.pone.0166367 (PMC5112889; doi:10.1371/journal.pone.0166367)
Supplement: S2 Table — (DOCX) [file pone.0166367.s004.docx]

**S2 Table. Excluded CPG information.**

| **Title** | **Year of publication** | **Rational** |
| --- | --- | --- |
| Rheumatoid arthritis: diagnostic and treatment [1] | 2002 | Older version. Latest version was selected: 2012 Brazilian Society of Rheumatology Consensus for the treatment of rheumatoid arthritis [2] |
| IV Brazilian guidelines for the management of asthma – 2006 [3] | 2006 | Older versions. Latest version was selected: Brazilian Guidelines for the management of asthma – 2012 [4] |
| III Brazilian consensus for the management of asthma – 2002 [5] | 2002 |  |
| I Brazilian consensus for the education of asthma - 1995 [6] | 1996 |  |
| Clinical protocol and therapeutic guidelines in Acute Coronary Syndromes [7] | 2011 | Not listed; this CPG does not focus on a chronic coronary syndrome |
| Summary of the II Brazilian guideline update on acute heart failure 2009/2011 [8] | 2012 |  |
| Review of the guidelines of the Brazilian Medical Association for the treatment of depression (Full version) [9] | 2009 | Older version. Latest version was selected: Unipolar depression: treatment – 2011 [10] |
| Diagnosis and Treatment of Depression [11] | 2001 |  |
| Diabetes Mellitus: Insulin therapy [12] | 2005 | Older version. Latest version was selected: Type 2 Diabetes Mellitus: Insulinization [13] |
| V Brazilian Guidelines on Hypertension – 2006 [14] | 2006 | Older version. Latest version was selected: VI Brazilian Guidelines on Hypertension [15] |
| IV Brazilian Guidelines on Hypertension [16] | 2004 |  |
| III Brazilian Consensus of Hypertension  arterial [17] | 1999 |  |
| Osteoarthritis (arthrosis): treatment [18] | 2003 | Older version. Latest version was selected: Use of nonsteroidal anti-inflammatory drugs in Chronic Pain in Patients with Osteoarthritis (Osteoarthritis) - 2011 [19] |
| Brazilian consensus on osteoporosis [20] | 2002 | Older version. Latest version was selected: Osteoporosis: treatment – 2011 [21] |
| Guidelines on atrial fibrillation [22] | 2003 | Older version. Latest version was selected: Brazilian atrial fibrillation guidelines [23] |
| Guidelines of the Brazilian Society of Diabetes 2013-2014 [24] | 2014 | Older version. Latest version was selected: Guidelines of the Brazilian Society of Diabetes 2014-2015 [25] |
| Guidelines for the diagnosis and management of gastroesophageal reflux disease: an evidence-based consensus [26] | 2010 | Older version. Latest version was selected: Gastroesophageal reflux disease: pharmacological treatment – 2011 [27] |
| Gastroesophageal reflux: diagnosis and treatment [28] | 2003 |  |
| Chronic obstructive pulmonary disease [29] | 2001 | Older version. Latest version was selected: II Brazilian Consensus on Chronic obstructive pulmonary disease - COPD – 2004 [30] |
| III Brazilian Guidelines on Chronic Heart Failure [31] | 2009 | Older version. Latest version was selected: Updating of the Brazilian guideline for chronic heart failure – 2012 [32] |
| Review of II guidelines of the Brazilian Cardiology Society for the diagnosis and treatment of heart failure [33] | 2002 |  |
| IV Brazilian guidelines for dyslipidemia and atherosclerosis prevention [34] | 2007 | Older version. Latest version was selected: V Brazilian Guidelines on Dyslipidemias and Prevention of Atherosclerosis – 2013 [35] |
| Atherosclerosis Prevention - Dyslipidemia [36] | 2001 |  |
| III Brazilian guidelines on dyslipidemia and guideline of atherosclerosis prevention [37] | 2001 |  |
| II Brazilian Consensus on Dyslipidemia [38] | 2000 |  |
| Second Brazilian Consensus Conference on Helicobacter pylori infection [39] | 2005 | Not listed disease as this CPG focused on *Helicobacter pylori* infection. |
| Treatment of Alzheimer's disease: recommendations and suggestions [40] | 2005 | Older version. Latest version was selected: Alzheimer's Disease: Prevention and Treatment - 2011[41] |
| I Brazilian position paper on antihypertensive drug combination [42] | 2014 | This is not a CPG |
| First Brazilian guidelines for familial hypercholesterolemia [43] | 2012 | Not listed; this CPG focused on familial hypercholesterolemia. |
| Osteoporosis induced by glucocorticoids:  prevention and treatment [44] | 2011 | Not listed; this CPG focused on glucocorticoid use only. |
| 3rd Brazilian Consensus on *Helicobacter pylori* [45] | 2013 | Not listed; this CPG focused on *Helicobacter pylori* infection. |

^CPG: clinical practice guideline.^

**References**

1. Brazilian Society of Rheumatology. [Rheumatoid Arthritis: Diagnostic and Treatment]. In: Brazilian Medical Association [Internet]. 2002 [cited 30 Oct 2015] p. 15. Available: http://www.projetodiretrizes.org.br/projeto_diretrizes/015.pdf

2. Mota LMH da, Cruz BA, Brenol CV, Pereira IA, Rezende-Fronza LS, Bertolo MB, et al. 2012 Brazilian Society of Rheumatology Consensus for the treatment of rheumatoid arthritis. Rev Bras Reumatol. 2012;52: 152–74. doi:10.1590/S0482-50042012000200002

3. Brazilian Society of Pneumology and Tisiology. [IV Brazilian Guidelines for the management of asthma]. J Bras Pneumol. 2006;32: s447–s474. doi:10.1590/S1806-37132006001100002

4. Brazilian Society of Pneumology and Tisiology. [Brazilian Guidelines for the management of asthma - 2012]. J Pneumol. 2012;38: S1–46. Available: http://www.sbpt.org.br/downloads/arquivos/COM_ASMA/SBPT_DIRETRIZES_MANEJO_ASMA_SBPT_2012.pdf

5. Brazilian Society of Pneumology and Tisiology. III Brazilian consensus for the management of asthma – 2002. J Pneumol. 2002;28: S6–S51. doi:10.1590/S0102-35862002000700004

6. Brazilian Society of Pneumology and Tisiology. [I Brazilian consensus for the education of asthma - 1995 ]. J Pneumol. 1996;22: 1–25.

7. Brazilian Ministry of Health. [Clinical protocol and therapeutic guidelines in Acute Coronary Syndromes]. In: Clinical protocol and therapeutic guidelines [Internet]. 2011 [cited 30 Oct 2015]. Available: http://portalsaude.saude.gov.br/index.php/o-ministerio/principal/leia-mais-o-ministerio/840-sctie-raiz/daf-raiz/cgceaf-raiz/cgceaf/l3-cgceaf/11646-pcdt

8. Montera MW, Pereira SB, Colafranceschi AS, Almeida DR de, Tinoco EM, Rocha RM, et al. Summary of the II Brazilian guideline update on acute heart failure 2009/2011. Arq Bras Cardiol. Brazil; 2012;98: 375–383. doi:http://dx.doi.org/10.1590/S0066-782X2012000500001

9. Fleck MP, Berlim MT, Lafer B, Sougey EB, Del Porto JA, Brasil MA, et al. Review of the guidelines of the Brazilian Medical Association for the treatment of depression. Rev Bras Psiquiatr. 2009;31: S7–S17. doi:S1516-44462009000500003 [pii]

10. Fleck M, Chagas M, Guapo V, Brasil M, Giribela A, Toledo S, et al. [Unipolar depression: treatment]. In: Brazilian Medical Association [Internet]. 2011 [cited 30 Oct 2015]. Available: http://www.projetodiretrizes.org.br/ans/diretrizes/depressao_unipolar-Tratamento.pdf

11. Fleck, MPA; Lafer, B; Sougey E et al. [Diagnosis and Treatment of Depression]. In: Brazilian Medical Association [Internet]. 2001 [cited 30 Oct 2015]. Available: http://www.projetodiretrizes.org.br/projeto_diretrizes/036.pdf

12. Hissa MN. [Diabetes Mellitus: Insulin therapy]. In: Brazilian Medical Association [Internet]. 2005 [cited 30 Oct 2015]. Available: http://www.projetodiretrizes.org.br/4_volume/07-Diabetes-I.pdf

13. Macedo G, Moura F, Soriano EA, Ribas DF AN. [Type 2 Diabetes Mellitus: Insulinization]. In: Brazilian Medical Association [Internet]. 2011 [cited 30 Oct 2015]. Available: http://www.projetodiretrizes.org.br/diretrizes10/diabetes_mellitus_tipo_2_insulinizacao.pdf

14. Brazilian Society of Cardiology, Brazilian Society of Hypertension, Brazilian Society of Nephrology. [V Brazilian Guidelines in Arterial Hypertension]. Arq Bras Cardiol. 2007;89: 121–157. doi:10.1590/S0066-782X2007001500012

15. Brazilian Society of Cardiology, Brazilian Society of Hypertension, Brazilian Society of Nephrology. [VI Brazilian Guidelines on Hypertension]. Arq Bras Cardiol. 2010;95: 1–51. doi:10.1590/S0066-782X2010001700001

16. Mion Jr. D, Gomes MAM, Nobre F, Amodeo C, Kohlmann Jr. O, Praxedes JN, et al. [IV Brazilian Guidelines on Hypertension]. Arq Bras Cardiol. 2004;82: 7–14. doi:10.1590/S0066-782X2004001000004

17. Kohlmann Jr. O, Costa Guimarães A, Carvalho MHC, Chaves Jr. H de C, Machado CA, Praxedes JN, et al. [III Brazilian Consensus on Hypertension]. Arq Bras Endocrinol Metab. 1999;43: 257–86. doi:10.1590/S0004-27301999000400004

18. Coimbra IB, Pastor EH, Greve JMD, Puccinelli MLC, Fuller R, Cavalcanti FS, Maciel FMB HE. [Osteoarthritis (arthrosis): treatment]. In: Brazilian Medical Association [Internet]. 2003 [cited 30 Oct 2015]. Available: http://www.projetodiretrizes.org.br/projeto_diretrizes/077.pdf

19. Coimbra I, Ferreira B, Coimbra A, Anderson M, Andrada N. [Use of nonsteroidal antiinflammatory drugs in chronic pain in patients with osteoarthritis (osteoarthrosis)]. In: Brazilian Medical Association [Internet]. 2011. Available: http://www.projetodiretrizes.org.br/ans/diretrizes/uso_dos_anti-inflamatorios_nao_hormonais_na_dor_cronica_em_pacientes_com_osteoartrite_(osteoartrose).pdf

20. Pinto Neto AMP, Soares A, Urbanetz AA, Souza ACDAE, Ferrari AEM, Amaral B, et al. [Brazilian consensus on osteoporosis 2002]. Rev Bras Reumatol. 2002;42: 343–54.

21. Cunha, EP; Steiner, ML; Strufaldi, R; Fernandes, C; Laurindo, IMM; Pereira, RMR; Simões R. [Osteoporosis: treatment]. In: Brazilian Medical Association [Internet]. 2011 [cited 30 Oct 2015]. Available: http://www.projetodiretrizes.org.br/ans/diretrizes/osteoporose-tratamento.pdf

22. Martinelli Filho M, Moreira DAR, Lorga AM, Sosa E, Atié J, Pimenta J, et al. [Guidelines on Atrial Fibrillation]. Arq Bras Cardiol. Arquivos Brasileiros de Cardiologia; 2003;81: 2–24. doi:10.1590/S0066-782X2003002000002

23. Zimerman LI, Fenelon G, Martinelli-Filho M, Grupi C, Atié J, Lorga-Filho A. [Brazilian atrial fibrillation guidelines]. Arq Bras Cardiol. 2009;92: 1–39.

24. Brazilian Society of Diabetes. [Guidelines of the Brazilian Society of Diabetes 2013-2014]. São Paulo: AC Farmacêutica; 2014.

25. Brazilian Society of Diabetes. [Guidelines of the Brazilian Society of Diabetes 2014-2015]. São Paulo: AC Farmacêutica; 2015.

26. Moraes-Filho JPP, Navarro-Rodriguez T, Barbuti R, Eisig J, Chinzon D, Bernardo W, et al. Guidelines for the diagnosis and management of gastroesophageal reflux disease: An evidence-based consensus. Arq Gastroenterol. 2010;47: 99–115. doi:10.1590/S0004-28032010000100017

27. Carvalhaes A, Ferrari A, Magalhães A, Nasy A, Paula e Silva C, Hashimoto C, et al. [Gastroesophageal reflux disease: pharmacological treatment]. In: Brazilian Medical Association [Internet]. 2011 [cited 30 Oct 2015]. Available: http://www.projetodiretrizes.org.br/ans/diretrizes/doenca_do_refluxo_gastroesofagico-tratamento_farmacologico.pdf

28. Chinzon D, Rossini ARA KB, Navarro-Rodrigues T, Barbuti RC HC, Eisig JN M-FJ. [Gastroesophageal Reflux: Diagnosis and Treatment]. In: Brazilian Medical Association [Internet]. 2003 [cited 30 Oct 2015]. Available: http://www.projetodiretrizes.org.br/projeto_diretrizes/084.pdf

29. Cukier A, Franco C, Barbas C, Kirchenchtejn C, Folador E, Jatene F, et al. [Chronic Obstructive Pulmonary Disease]. In: Brazilian Medical Association [Internet]. 2001 [cited 30 Oct 2015]. Available: http://www.projetodiretrizes.org.br/projeto_diretrizes/042.pdf

30. Brazilian Society of Pneumology and Tisiology. [II Brazilian Consensus on Chronic obstructive pulmonary disease - COPD - 2004]. J Bras Pneumol. 2004;30: S1–S42. doi:ISSN 1806-3713

31. Bocchi EA, Braga FGM, Ferreira SMA, Rohde LEP, Oliveira WA de, Almeida DR de, et al. [III Brazilian Guidelines on Chronic Heart Failure]. Arq Bras Cardiol. Arquivos Brasileiros de Cardiologia; 2009;93: 3–70. doi:S0066-782X2009002000001 [pii]

32. Bocchi EA, Marcondes-Braga FG, Bacal F, Ferraz AS, Albuquerque D, Rodrigues D, et al. [Updating of the Brazilian guideline for chronic heart failure - 2012]. Arq Bras Cardiol. 2012;98: 1–33. doi:http://dx.doi.org/10.1590/S0066-782X2012000700001

33. Guimarães JI, Mesquita ET, Bocchi EA, Vilas-Boas F, Guimaräes JI, Mesquita ET, et al. [Review of II guidelines of the Brazilian Cardiology Society for the diagnosis and treatment of heart failure]. Arq Bras Cardiol. 2002;79: 1–30. doi:10.1590/S0066-782X2002001800001

34. Sposito AC, Caramelli B, Fonseca FAH, Bertolami MC, Afiune Neto A, Souza AD, et al. [IV Brazilian Guideline for Dyslipidemia and Atherosclerosis prevention: Department of Atherosclerosis of Brazilian Society of Cardiology]. Arq Bras Cardiol. 2007;88: 2–19. doi:10.1590/S0066-782X2007000700002

35. Xavier HT, Izar MC, Faria Neto JR, Assad MH, Rocha VZ, Sposito AC, et al. [V Brazilian Guidelines on Dyslipidemias and Prevention of Atherosclerosis]. Arq Bras Cardiol. 2013;101: 1–20. doi:10.5935/abc.2013S010

36. Santos RD, Giannini SD, Moriguchi EH, Fonseca FH Participantes: Pereira A, Afiúne Neto A, Souza AD, Avezum A, Faludi AA, Spósito AC, Loures-Vale AA, Lottenberg AM, Chagas ACP, Mansur AP, Guimarães A, Duncan B, Caramelli B, Mota CCC, Polanczyl C, Scherr C, C, Sociedade Brasileira de Cardiologia. [Atherosclerosis Prevention - Dyslipidemia]. In: Brazilian Medical Association [Internet]. 2001 [cited 30 Oct 2015]. Available: http://projetodiretrizes.org.br/projeto_diretrizes/040.pdf

37. Sposito AC, Caramelli B, Fonseca FA, Bertolami MC, Afiune Neto A, Souza AD et al; [III Brazilian Guidelines on Dyslipidemias and Guideline of Atherosclerosis Prevention from Atherosclerosis Department of Sociedade Brasileira de Cardiologia]. Arq Bras Cardiol. 2001;77: 1–48. doi:http://dx.doi.org/10.1590/S0066-782X2001001500001

38. Santos Filho RD dos. [II Brazilian consensus about dyslipidemia]. Rev Assoc Med Bras. 2000;46: 307. doi:10.1590/S0104-42302000000400025

39. Luis Coelho SZ. [Second Brazilian Consensus Conference on Helicobacter pylori infection]. Arq Gastroenterol. 2005;42: 128–32. doi:http://dx.doi.org/10.1590/S0004-28032005000200012

40. Nitrini R, Caramelli P, Bottino CM de C, Damasceno BP, Brucki SMD, Anghinah R. [Treatment of Alzheimers Disease: recommendations and suggestions of the Scientific Department of Cognitive Neurology and Aging of the Brazilian Academy of Neurology]. Arq Neuro-Psiquiatr. 2005;63: 1104–12. doi:http://dx.doi.org/10.1590/S0004-282X2005000600035

41. Lopes L, Araújo L, Chaves M, Imamura M, Okamoto I, Ramos A, et al. [Alzheimer’s Disease: Prevention and Treatment]. In: Brazilian Medical Association [Internet]. 2011 [cited 30 Oct 2015]. Available: http://www.projetodiretrizes.org.br/ans/diretrizes/doenca_de_alzheimer-prevencao_e_tratamento.pdf

42. Povoa R, Barroso WS, Brandao AA, Jardim PCV, Barroso O, Passarelli OJ, et al. [I Brazilian Position Paper on Antihypertensive Drug Combination]. Arq Bras Cardiol. 2014;102: 203–10. doi:10.5935/abc.20140023

43. Santos RDR, Gagliardi ACM, Xavier HT, Casella Filho A, Araújo DB, Cesena FY, et al. [First Brazilian Guidelines for Familial Hypercholesterolemia]. Arq Bras Cardiol. 2012;99: 1–28. doi:10.5935/abc.20120202

44. Pereira R, Paula A, Zerbini C, Domiciano D, Gonçalves H, Danowski J, et al. [Osteoporosis induced by glucocorticoids: prevention and treatment]. In: Brazilian Medical Association [Internet]. 2011 [cited 30 Oct 2015]. Available: http://www.projetodiretrizes.org.br/projeto_diretrizes/osteoporose_induzida_por_glicocorticoide.pdf

45. Coelho LG, Maguinilk I, Zaterka S, Parente JM, do Carmo Friche Passos M, Moraes-Filho JPP. 3rd Brazilian Consensus on Helicobacter pylori. Arq Gastroenterol. 2013;50: 81–96. doi:10.1590/S0004-28032013005000001
